# Contrast-enhanced ultrasound of the small organs in children

**DOI:** 10.1007/s00247-021-05006-x

**Published:** 2021-04-08

**Authors:** Maciej Piskunowicz, Susan J. Back, Kassa Darge, Paul D. Humphries, Jörg Jüngert, Damjana Ključevšek, Norbert Lorenz, Hans-Joachim Mentzel, Judy H. Squires, Dean Y. Huang

**Affiliations:** 1 Department of Radiology, Medical University of Gdansk, M. Sklodowskiej-Curie 3a Street, 80-210 Gdansk, Poland; 2 Department of Radiology, Children’s Hospital of Philadelphia, Perelman School of Medicine, University of Pennsylvania, Philadelphia, PA USA; 3 Department of Radiology, Great Ormond Street Hospital for Children NHS Trust, London, UK; 4 Department of Pediatrics, Friedrich-Alexander-University Erlangen-Nuremberg, Erlangen, Germany; 5 Department of Radiology, University Children’s Hospital Ljubljana, Ljubljana, Slovenia; 6 Children’s Hospital, Dresden Municipal Hospital, Teaching-Hospital of Technical University Dresden, Dresden, Germany; 7 Section of Pediatric Radiology, Institute of Diagnostic and Interventional Radiology, University Hospital, Jena, Germany; 8 Department of Radiology, University of Pittsburgh Medical Center, Children’s Hospital of Pittsburgh, Pittsburgh, PA USA; 9 Department of Radiology, King’s College Hospital, Denmark Hill, London, UK

**Keywords:** Children, Contrast-enhanced ultrasound, Lymph nodes, Ovary, Testis, Thyroid, Ultrasound, Ultrasound contrast agents, Uterus

## Abstract

In pediatric and adult populations, intravenous contrast-enhanced ultrasound (CEUS) remains off-label for imaging of organs other than the liver and heart. This limited scope inhibits potential benefits of the new modality from a more widespread utilization. Yet, CEUS is potentially useful for imaging small organs such as the thyroid gland, lymph nodes, testes, ovaries and uterus, with all having locations and vasculature favorable for this type of examination. In the adult population, the utility of CEUS has been demonstrated in a growing number of studies for the evaluation of these small organs. The aim of this article is to present a review of pediatric CEUS of the thyroid gland, lymph nodes, testes, ovaries and uterus as well as to draw from the adult literature indications for possible applications in children.

**Supplementary Information:**

The online version contains supplementary material available at 10.1007/s00247-021-05006-x.

## Introduction

Ultrasound (US) is the imaging method of choice to evaluate superficial and deeply located small organs such as the thyroid gland, lymph nodes, testes, ovaries and uterus. Diagnosis of various pathologies in these organs is frequently established based on gray-scale and color Doppler US examination. Yet, there is growing experience with the use of contrast-enhanced ultrasound (CEUS) as an adjunct problem-solving tool or part of a multiparametric imaging algorithm in several clinical contexts. In their 2018 imaging recommendations, the European Federation of Societies for Ultrasound in Medicine and Biology (EFSUMB) for the first time underlined the potential utility of CEUS for evaluating all of these small organs in adults [1]. In children, there is limited experience regarding the use of CEUS for evaluating testicular infarction and ovarian torsion [2, 3]. However, the cumulative experience from the established CEUS applications in various organs and body parts so far makes it clear that there is a potential role for the use of CEUS in the evaluation of these small organs in children. In this review article, we present the current experience, examination technique and main imaging findings for CEUS in the evaluation of the thyroid gland, lymph nodes, testes, ovaries and uterus. We highlight the possible benefits of these applications, focusing on children based on authors’ experience and also extrapolating knowledge from available publications, which are mostly in the adult population.

## Contrast-enhanced ultrasound examination technique

### Patient preparation

No specific patient preparation is required for CEUS imaging of the thyroid gland, lymph nodes and testes. Optimal transabdominal CEUS examination of the uterus and ovaries requires a well distended urinary bladder, which serves as an acoustic window for adequate visualization of these organs, similar to gray-scale US. Therefore, oral fluid intake is recommended prior to the examination. In an urgent setting, such as the evaluation of ovarian torsion, it might not be possible to wait for physiological bladder filling. In such cases, some centers place a catheter into the urinary bladder and fill it with normal saline prior to the US examination.

The recommended intravenous (IV) catheters for the ultrasound contrast agent (UCA) injection are peripherally inserted 18- to 22-gauge (G) IV catheters; however, for newborns 24- to 26-G catheters can be used as well.

### Ultrasound contrast agent dose

The United States Food and Drug Administration (FDA) approved Lumason (Bracco Diagnostics Inc., Monroe Township, NJ) for pediatric liver and cardiac IV CEUS examinations. The approved dosing is 0.03 mL/kg, up to a maximum of 2.4 mL and 2 mL per injection for liver and cardiac applications, respectively. Lumason is marketed outside the United States as SonoVue (Bracco Imaging SpA, Milan, Italy). Other UCAs that have been used for IV CEUS applications in children and adults in the United States are Optison (GE Healthcare, Princeton, NJ) and Definity (Lantheus Medical Imaging, North Billerica, MA). Currently, use of all UCAs to examine small organs in adults and children are off-label indications, and therefore a range of doses have been reported in the literature.

Prior to FDA approval of Lumason, various age-based or weight-based dosing regimens were used for IV CEUS examinations of solid abdominal organs for characterizing indeterminate focal lesions and detecting traumatic injuries. Some of the most commonly reported dosing regimens are: 1.2–2.4 mL SonoVue for children ≥1 year old and 0.6 mL SonoVue for children <1 year old; 0.1 mL/kg SonoVue for children weighing up to 24 kg and fixed dose of 2.4 mL for those weighing more than 24 kg; or age-based dose according to the formula: volume of UCA (mL) = age (in years)/20 for organs other than liver [4–6].

Determining optimal UCA dosing in small organs, particularly in children, requires special consideration. In our experience, a UCA dose of 0.03–0.10 mL/kg of SonoVue/Lumason is likely to be sufficient for pediatric small organs CEUS imaging, though larger doses can be used when higher-frequency transducers are used. Two case reports of IV CEUS in children, reported use of 0.6 mL of SonoVue for imaging of the ovaries in a 6-year-old girl and 2 mL of SonoVue for imaging of the testis in an adolescent boy [2, 3].

Bolus injection doses in adults are also higher when evaluating these small organs in comparison to those used for the adult liver. When examining the adult thyroid, CEUS doses of 1.0–4.8 mL per bolus injection of SonoVue have been used [7–13]. Lymph node CEUS has been performed with bolus injections ranging from 1.5 to 4.8 mL [14–16]. For adult testicular CEUS examination, a bolus injection of up to 4.8 mL of SonoVue/Lumason is described [17–19]. Published data for CEUS examination of the ovaries and uterus in women using transvaginal transducers reported bolus injections from 2.4 mL to 4.8 mL [20, 21].

### Ultrasound transducers

For small superficial organs (thyroid, lymph nodes, testis) high-resolution, high-frequency (i.e. >10 MHz) linear transducers should be used. The ovaries and uterus, especially in children and adolescents, can be scanned with convex transducers with frequencies appropriate for the child’s body habitus (frequency range 1–9 MHz). In this age group, intracavitary transducers do not play a significant role.

## Thyroid gland

Ultrasound examination of the thyroid gland is performed for evaluating focal lesions or diffuse parenchymal abnormalities. Imaging characteristics of thyroid lesions are described and are well established for gray-scale US. Features include size, shape, borders, internal echotexture in comparison to adjacent normal thyroid tissue, presence of calcifications as well as Doppler imaging flow patterns. Strain and shear-wave elastography are also increasingly applied as part of noninvasive advanced US applications to quantify tissue stiffness.

**Fig. 1:**
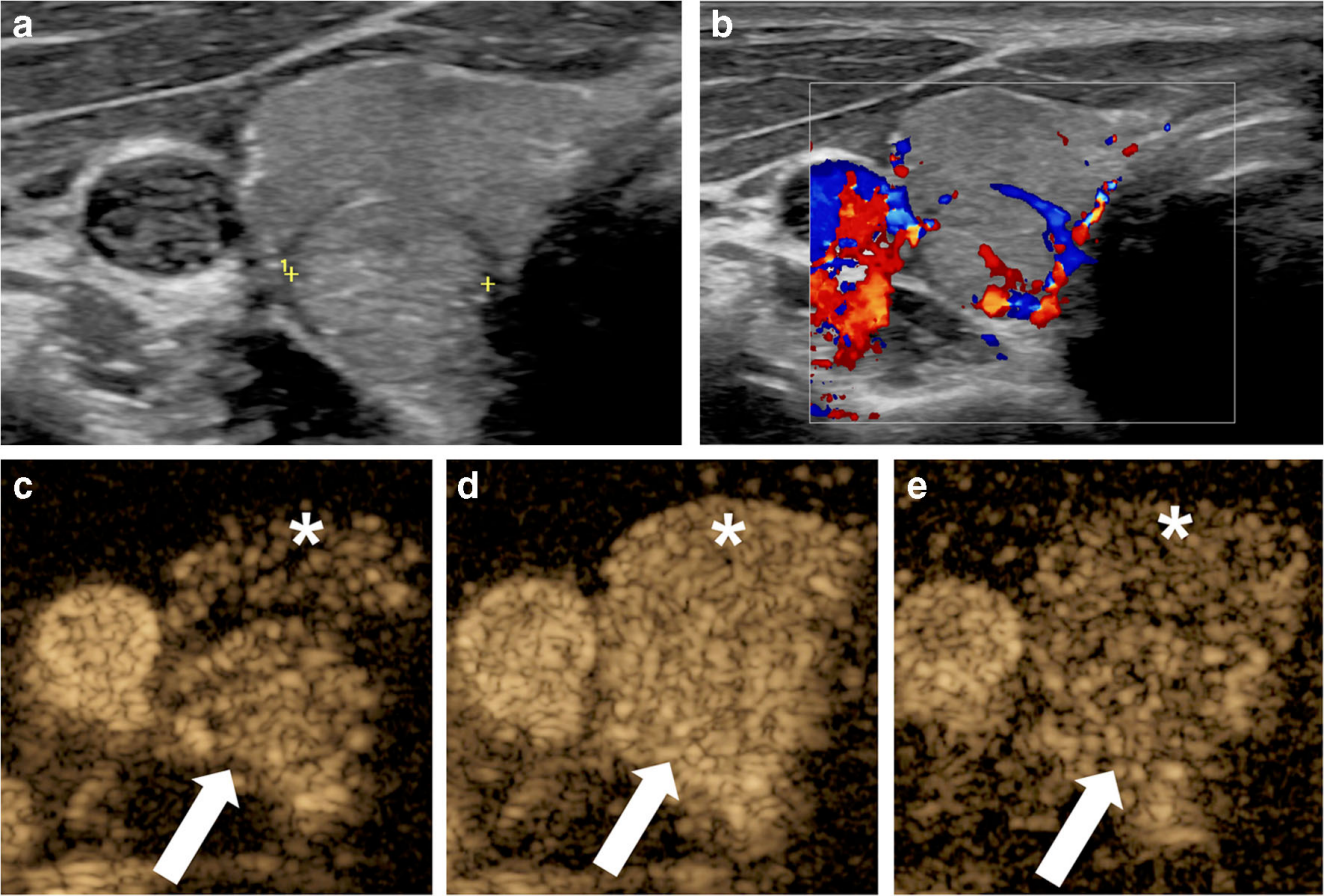
Right thyroid nodule that was incidentally detected on a CT scan of the cervical spine in a 17-year-old boy. The CT was obtained following a motor vehicle collision. **a** Transverse gray-scale ultrasound (US) of the right thyroid shows a well-circumscribed, solid, iso- to slightly hyperechoic nodule in the lower pole (*calipers*). **b** Transverse color Doppler US of the right thyroid nodule demonstrates predominantly peripheral vascularity. **c–e** Transverse contrast-enhanced ultrasound (CEUS) of the right thyroid lobe. Contrast-only mode. There is homogeneous enhancement of the nodule (*arrow*), which is slightly hyperenhancing to the overlying thyroid parenchyma (*asterisk*) in the arterial phase (**c**) and iso-enhancing in the parenchymal (**d**) and delayed (**e**) phases. Overall this is a benign CEUS enhancement pattern. The diagnosis following biopsy was a benign follicular adenoma, oncocytic type (Hurthle cell adenoma)

Routine application of IV CEUS is not well established for thyroid. Although numerous original studies and meta-analyses about the use and diagnostic value of IV CEUS for characterizing nodules in adults do exist, the 2018 EFSUMB guidelines describe the use of CEUS for characterizing thyroid nodules as an active field of clinical research, but at present it is not recommended for routine clinical use [1, 8, 22–24]. The reason for this limitation is the high variability in CEUS diagnostic performance because of the overlap in the enhancement features between typical or atypical benign and malignant thyroid nodules [25]. A couple of studies describe the application in autoimmune Hashimoto inflammation of the thyroid gland [24, 26]. In children, there are no reports yet on the application of CEUS for evaluating macro- and microvascularization in the thyroid gland (Fig. 1).

### Thyroid nodules

By palpation, the incidence of thyroid nodules in the adult population is approximately 4–7% [25]. However, that number increases up to 30% with the use of high-resolution US of the thyroid gland in clinical practice [11, 25]. The prevalence of thyroid nodules in children ranges between 1% to 2% and is significantly lower compared to adults [27]. Most nodules in adults are benign, with fewer than 5% classified as malignant [28]. However, in children thyroid nodules carry a far greater risk of harboring malignancy than in adults, at a rate of approximately 26.4% [27].

In the CEUS literature, thyroid nodules can be characterized and differentiated by qualitative and quantitative CEUS imaging parameters. Qualitative parameters describe nodule perfusion compared to the surrounding thyroid tissue and include observations of enhancement such as intensity, homogeneity, vascular distribution pattern and rate of contrast uptake [24, 25, 29]. The main CEUS features that are highly suspicious for malignancy are hypoenhancement of the nodule throughout the enhancement phases and heterogeneous enhancement [9, 30]. In one adult study, six qualitative CEUS features were considered significant for predicting malignant thyroid nodules: slow entry of contrast agents during the enhancement phase (odds ratio [OR]=15.6, *P*=0 .001), slow time-to-peak (OR=7.4, *P*=0.002), non-uniform enhancement (OR=10.1, *P*=0.023), irregular enhancement pattern (OR=36.2, *P*=0.002), unclear enhancement boundary (OR=25.3, *P*=0.012) and absence of ring-like enhancement (OR=25.3, *P*=0.004) [25]. On the contrary, uniform enhancement and ring enhancement are typical findings for predicting benignity [31]. However, the CEUS enhancement patterns of both malignant and benign nodules can overlap [29]. Because of the subjectivity in this assessment, a quantitative approach has been the subject of much research. Quantitative CEUS with time-intensity curve analysis can be performed following image acquisition using special software programs embedded on the US unit or on separate computers. This analysis requires that regions of interest be placed in the nodule and the adjacent normal tissue. Imaging parameters obtained from time-intensity curves including wash-in-slope, peak intensity, time-to-peak, area under the curve (AUC), mean transit time, and washout time have great potential to provide objective information for differentiating benign and malignant thyroid nodules [9]. In quantitative CEUS evaluations, later arrival time of contrast, later time-to-peak with earlier start of the washout phase, lower peak intensity and overall smaller AUC were strongly associated with malignancy [9]. Some adult studies, however, showed differences in CEUS quantitative parameters between small (<1 cm) and large (>1 cm) malignant thyroid nodules [9, 30]. Specifically, larger malignant nodules had a steeper slope of the wash-in curve, faster time-to-peak and slightly higher peak intensity compared with smaller malignant nodules. This could be because in smaller malignant nodules neoangiogenesis is incomplete, whereas in larger malignant nodules aberrant neo vessels result in more blood flow. Larger malignant nodules tend to have areas of necrosis, calcifications and fibrosis that diminish blood flow compared to benign nodules [9]. Still, as further validation of these CEUS parameters for thyroid nodules continues, they should be interpreted only with the use of clinical and laboratory findings, as well as with elastography.

At the moment, no data exist about the use and importance of CEUS in thyroid applications in children. The majority of adult criteria can likely be applied to children when selecting nodules for fine-needle aspiration, with the following provisions in pediatric patients: gray-scale US pattern and clinical context including history should be considered more than size, intrathyroidal thymic tissue might mimic a suspicious nodule, and diffuse sclerosing variant of papillary thyroid cancer presents with non-nodular diffuse infiltration associated with microcalcifications (“snowstorm”) [32]. More experience with pediatric thyroid CEUS is needed to understand its role in this population.

### Thyroiditis

Hashimoto thyroiditis is an increasingly prevalent autoimmune disease. Recently, it has been shown that Hashimoto thyroiditis is associated with a greater risk of thyroid cancer [33, 34]. Diagnosis of Hashimoto thyroiditis often relies on clinical presentation and laboratory findings, as well as results from gray-scale and color Doppler US. However, at times fine-needle aspiration is indicated, especially when thyroiditis co-exists with nodules [35]. Using CEUS to assess thyroid nodules might help to decrease the number of fine-needle aspirations in these patients [24, 26]. In one study, malignant papillary thyroid carcinomas in patients with Hashimoto thyroiditis tended to exhibit hypoenhancement compared to adjacent parenchyma [24]. Using time-intensity curve analysis, the authors generated ratios of parameters from regions of interest within the nodule compared to the thyroid parenchyma and showed that the peak intensity ratio (peak intensity nodule/peak intensity parenchyma) and AUC ratio (AUC nodule/AUC parenchyma) tended to be <1 in malignant compared to benign lesions [24]. In the future, CEUS evaluation of tissue microvascularity might allow quantification of Hashimoto thyroiditis activity and differentiation from other diffuse thyroid disorders such as Graves disease.

## Lymph nodes

Family physicians and pediatricians often encounter superficial swellings and bumps. In these cases, US is frequently employed as a first-line tool to evaluate palpable findings, identify and assess lymph nodes, or differentiate among other causes of palpable lumps. Therefore, US examination of lymph nodes in children is a very common scenario.

There are well-established features on gray-scale and color/power Doppler US that can be used to assess the morphology and vascularity of individual lymph nodes. Some commonly reported characteristics of benign lymph nodes are a length-to-transverse diameter ratio (Solbiati index) greater than 2, the presence of a centrally located echogenic hilum surrounded evenly by a peripheral homogeneously hypoechoic cortex with smooth borders [36]. Gray-scale US findings suggestive of malignancy include lymph nodes with round shape (length-to-diameter ratio of less than 2), absence or distortion of the echogenic hilum and presence of focal intranodal hypoechoic or anechoic regions that represent areas of infiltration/necrosis. A global hypoechoic (i.e. pseudocystic) appearance of a lymph node is seen in cases of lymphoma infiltration [37]. Infection can appear similar to malignant nodes because of necrosis and abscess formation, which appears as hypoechoic regions on gray-scale US. On Doppler imaging, blood vessels in benign lymph nodes originate at the hilum with linear branches directed toward the cortex in a regular pattern. When inflamed there is increased blood flow within these normally distributed vessels. In both lymphoma and lymphadenitis, the pathological changes usually start from the center of the node. Therefore the hilar vascularity is usually preserved but augmented early on in most inflammatory nodes and those harboring lymphoma. Neovascularity that develops in lymph nodes with malignancy as well as those with tuberculosis tends to be in the periphery along the capsular contour as well. Centrally short-segment vessels appear cut-off, distorted or arranged in a chaotic pattern [36]. Perfusion defects can develop in areas of necrosis or infection. The resistive and pulsatility indices might also be elevated in cases of malignancy [16, 38]. Frequently, further management is guided by the clinical context in combination with conventional US findings, yet even in routine cases it is not uncommon to encounter equivocal US findings that require more in-depth evaluation.

The diagnostic performance of CEUS for the evaluation of superficial lymph nodes has not been reported in the pediatric literature. However, adult studies of IV CEUS with qualitative evaluation and, more recently, quantitative assessment of vascular flow kinetics derived from contrast injection have been performed to differentiate among reactive, inflammatory and metastatic superficial lymphadenopathy [16, 36, 39]. These studies showed that CEUS improves the conspicuity of the nodal perfusion pattern compared to vascular flow assessment with color/power Doppler US. CEUS imaging of a normal lymph node in early arterial phase shows hilar enhancement with the artery entering the hilum and spreading centrifugally. At that point, the whole lymph node rapidly enhances intensely and homogeneously. Lymph nodes can have homogeneous or non-homogeneous enhancement and have various intranodal vascular patterns on CEUS, similar to the lymph node Doppler US descriptions [40]. Lymph node architecture might be preserved early on in inflammation and lymphomatous infiltration. In lymphoma, new capillary circulation increases the blood flow to the node, producing a hyperenhancing CEUS appearance. Increased flow through normal hilar vessels in reactive lymph nodes results in hyperemia that can be difficult to distinguish from lymphoma [14, 37].

Qualitative CEUS adult studies coupled with time-intensity analysis have been performed in people with suspected metastatic lymphadenopathy, often in the setting of head and neck cancer. A variety of CEUS enhancement patterns for benign/reactive and malignant nodes have been described. In one study, intense but homogeneous enhancement was more commonly associated with reactive lymphadenopathy, whereas predominantly peripheral enhancement with perfusion defects or complete absence of enhancement were indicative of malignancy [41]. An early study using color Doppler US classified the intranodal vascular patterns into eight categories ranging from straight longitudinal hilar vessels with or without peripheral branches to significant alteration of nodal angioarchitecture with distortion and curved course of vessels, presence of aberrant or subcapsular vessels and complete absence of enhancement. The greater the degree of vascular distortion, the higher the risk of malignancy [40]. This classification system was later applied in a different study comparing CEUS to Doppler US in a cohort of adults with clinically enlarged superficial nodes scheduled for needle biopsy. In this study the addition of CEUS did not improve the diagnostic accuracy of Doppler techniques to detect more malignant lymph nodes [39]. In clinical practice, lymphadenitis can be observed with serial US imaging, and metastatic disease and tuberculosis can be diagnosed after core-needle biopsy, whereas lymphoma requires histological evaluation following surgical excision. One study in adults suggested that the intranodal contrast enhancement pattern could guide selection of which nodes should be biopsied, excised or observed with serial imaging [37]. This study demonstrated that CEUS can distinguish metastatic and tuberculous lymph nodes from lymphoma, which helps to guide the choice between biopsy and excision [37]. Two enhancement characteristics on CEUS were more indicative for the diagnosis of tuberculous or metastatic lymph nodes: peripheral perfusion in the early arterial phase and inhomogeneous spotted/cycle-like enhancement in the equilibrium phase. In cases of tuberculosis or metastatic infiltration, these signs have sensitivity and specificity of 80.4% and 81.2%, respectively [37]. On the contrary, progressive centrifugal enhancement pattern like a “snowstorm” in the arterial phase, with diffuse and uniform homogeneous enhancement in the parenchymal phase, was seen in the majority of patients with lymphoma.

Lymphoma is one of the most common childhood solid tumors and therefore is a clinically important entity, one that demands accurate detection and staging. Although the patterns described for metastatic nodes are consistent, the results from the adult literature of CEUS in lymphoma are somewhat conflicting. A wide variety of enhancement patterns have been described, with lymphoma mimicking both inflammatory and metastatic nodes [41, 42]. One study in adults with lymphoma described a rapid arterial hyperenhancement pattern that is either homogeneous or heterogeneous [43]. Another study in adults with superficial lymphadenopathy showed that intense homogeneous enhancement is more common in benign lymph nodes but can also be seen in lymphomas; inhomogeneous enhancement is typical for metastases, and complete absence of enhancement can be observed in lymphomas [44]. However, interpretation of these results is difficult because of the lack of a control group. At the present time, the role of CEUS for pediatric lymphoma is unclear.

Once again, quantitative CEUS imaging could be useful in this case, but the limited available data utilizing these methods for lymph node CEUS is conflicting. For example, the derived peak intensity, regional blood volume, time-to-peak and AUC were evaluated for a cohort of adults with suspected cervical lymph node metastases (histologically 32 benign, 29 malignant). The derived peak intensity and regional blood volume were significantly higher in benign nodes, while time-to-peak and AUC were similar in benign and malignant nodes [16]. But in contrast, another study found a significant difference between the time-to-peak and AUC of benign nodes vs. malignant nodes [44]. The times-to-peak of metastases and lymphomas were less than that of benign lymph nodes. Furthermore, because contrast enhancement lasted longer in benign lymph nodes, the AUC of benign lymph nodes was greater than that of malignant nodes and lymphomas [44]. Greater experience with more patients and exploration of additional parameters might support future use.

**Fig. 2:**
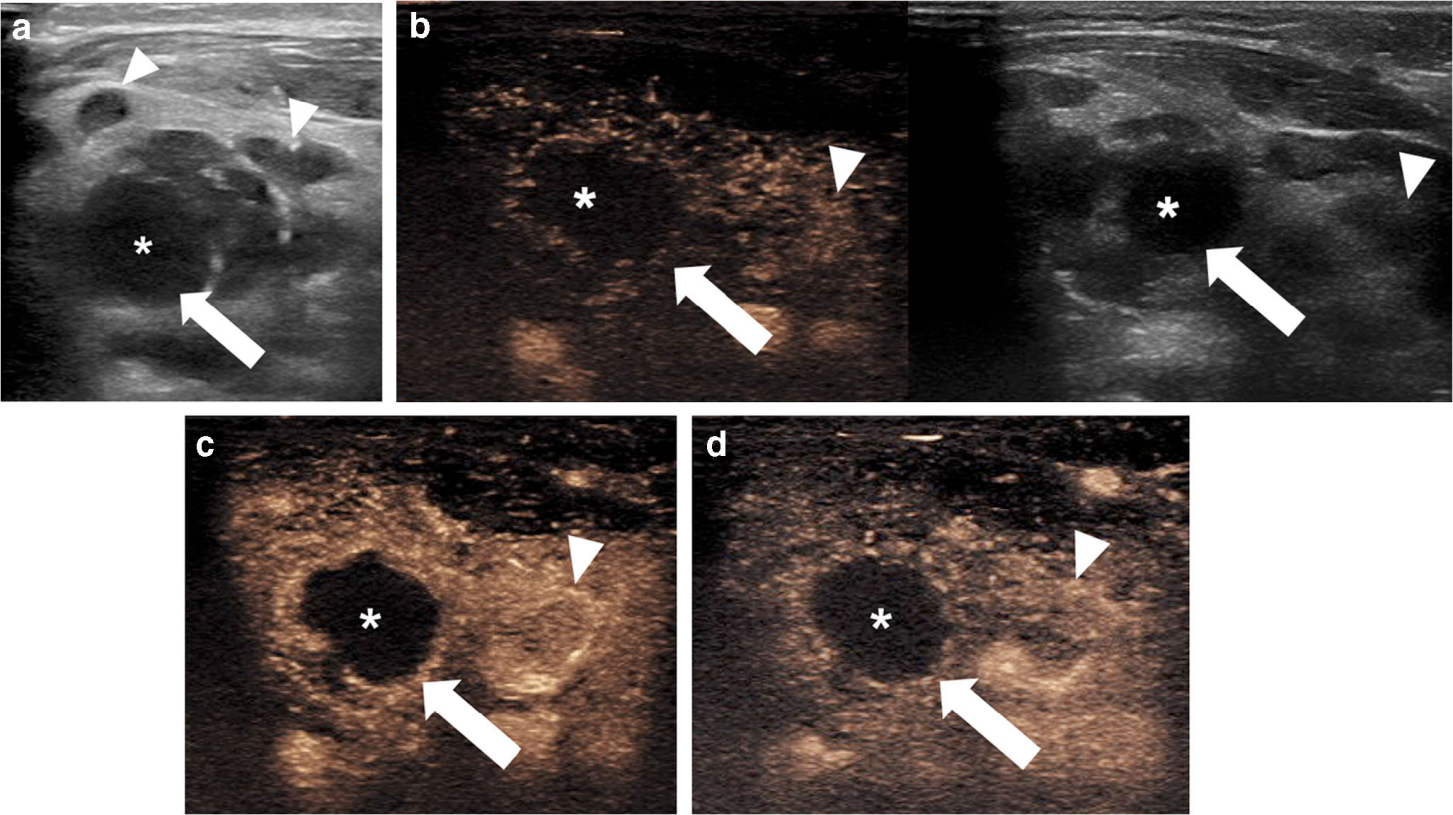
Severe right-side lymphadenitis colli in a 5-year-old girl. **a** Gray-scale US, transverse plane, shows multiple lymph nodes (*arrowheads*), one of which appears enlarged (*arrow*) with an anechoic area centrally (*asterisk*), suggestive of abscess formation. **b–d** Transverse contrast-enhanced ultrasound (CEUS) image with dual display of contrast (*left*) and gray-scale (*right*) modes and in contrast-only mode at 10 s (**b**), 15 s (**c**) and 39 s (**d**) post contrast injection. The central anechoic area of the lymph node (*asterisk*) demonstrates complete lack of enhancement corresponding to the purulent content. A hyperenhancing rim (*arrow*) is noted in the periphery corresponding to the adjacent inflamed tissue. An additional enlarged and abnormally enhancing lymph node is noted adjacent to the abscess (*arrowhead*)

**Fig. 3:**
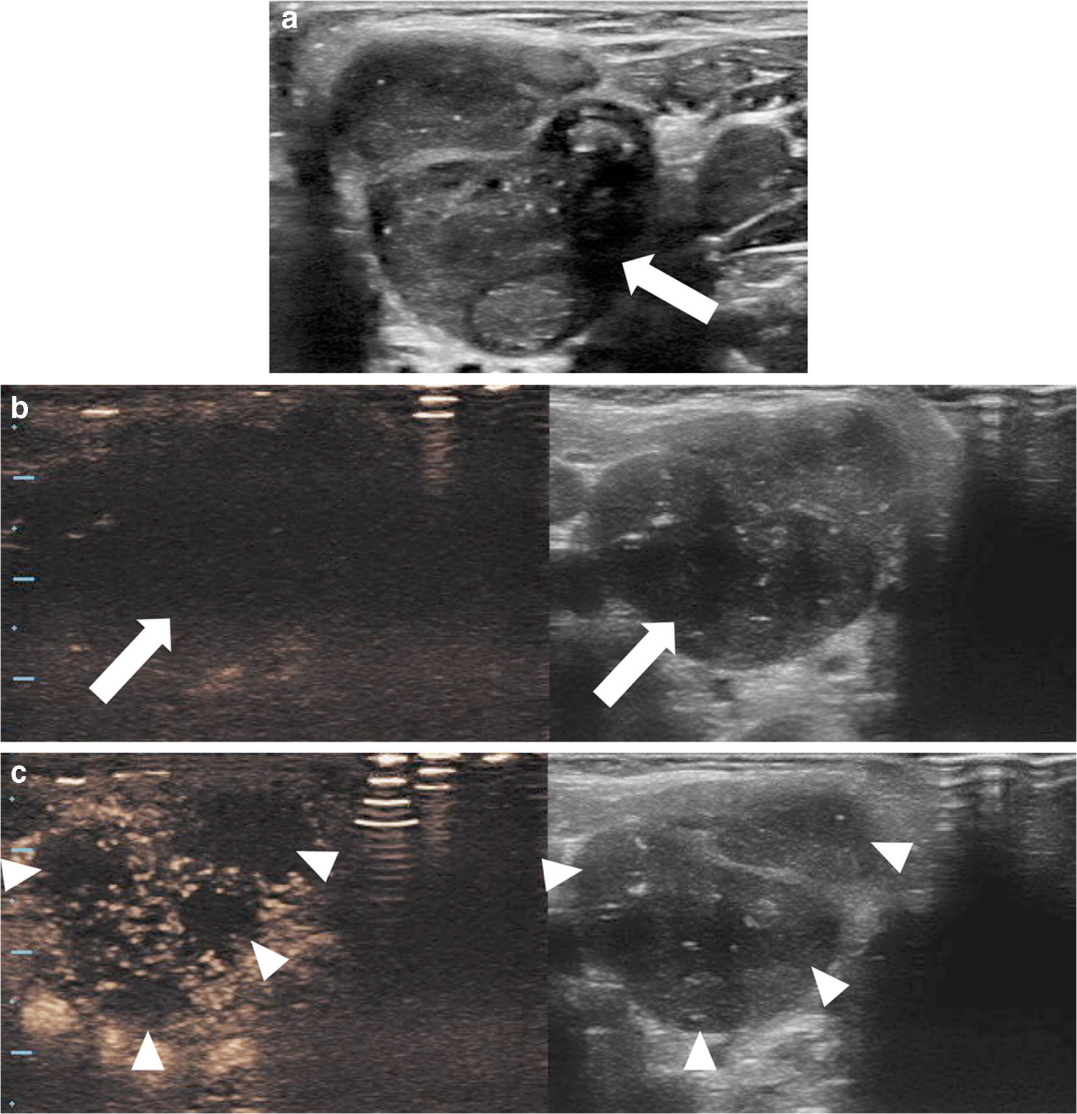
Atypical cervical mycobacteriosis in a 3-year-old girl. **a** Transverse gray-scale US demonstrates an enlarged lymph node (*arrow*) with heterogeneous internal reflectivity and presence of tiny hyperechoic punctate foci. **b, c** Transverse contrast-enhanced ultrasound (CEUS) with dual display of contrast (*left*) and gray-scale (*right*) modes. Ten seconds post contrast injection (**b**), no enhancement is seen within the lymph node (*arrow*). Fifteen seconds post contrast injection (**c**), there are multiple areas (*arrowheads*) of nonenhancement centrally within the lymph node, corresponding to caseating necrosis suggestive of atypical mycobacteriosis within the lymph node

Although the data are increasing in CEUS adult lymph node applications, it is difficult to extrapolate adult data to pediatric practice for two main reasons. First, pathologies are different in children, in whom carcinomatous metastases are exceedingly rare. Second, there is variance in the findings of published studies regarding the value of CEUS in the adult population [37, 39]. However, in our experience with children, CEUS might be a valuable alternative to MRI, which often requires anesthesia, for better delineation of abscessed lymph nodes in difficult cases, as well as in atypical lymph node tuberculosis (Figs. 2 and 3).

## Testes

Children with common pediatric testicular pathologies typically present with symptoms of acute scrotum (pain, swelling, redness), or a palpable finding. The etiology for these symptoms can be diverse [45]. US is unequivocally the first-line imaging modality for assessing pediatric scrotum. Although gray-scale and Doppler US techniques are reported to be well-suited for imaging the testis, several pitfalls remain in the evaluation of testicular pathology. Current literature indicates that because of its superior capability in assessing testicular parenchymal perfusion, CEUS could augment non-contrast evaluation of testes, including the most pertinent pediatric indications [18, 46–48].

**Fig. 4:**
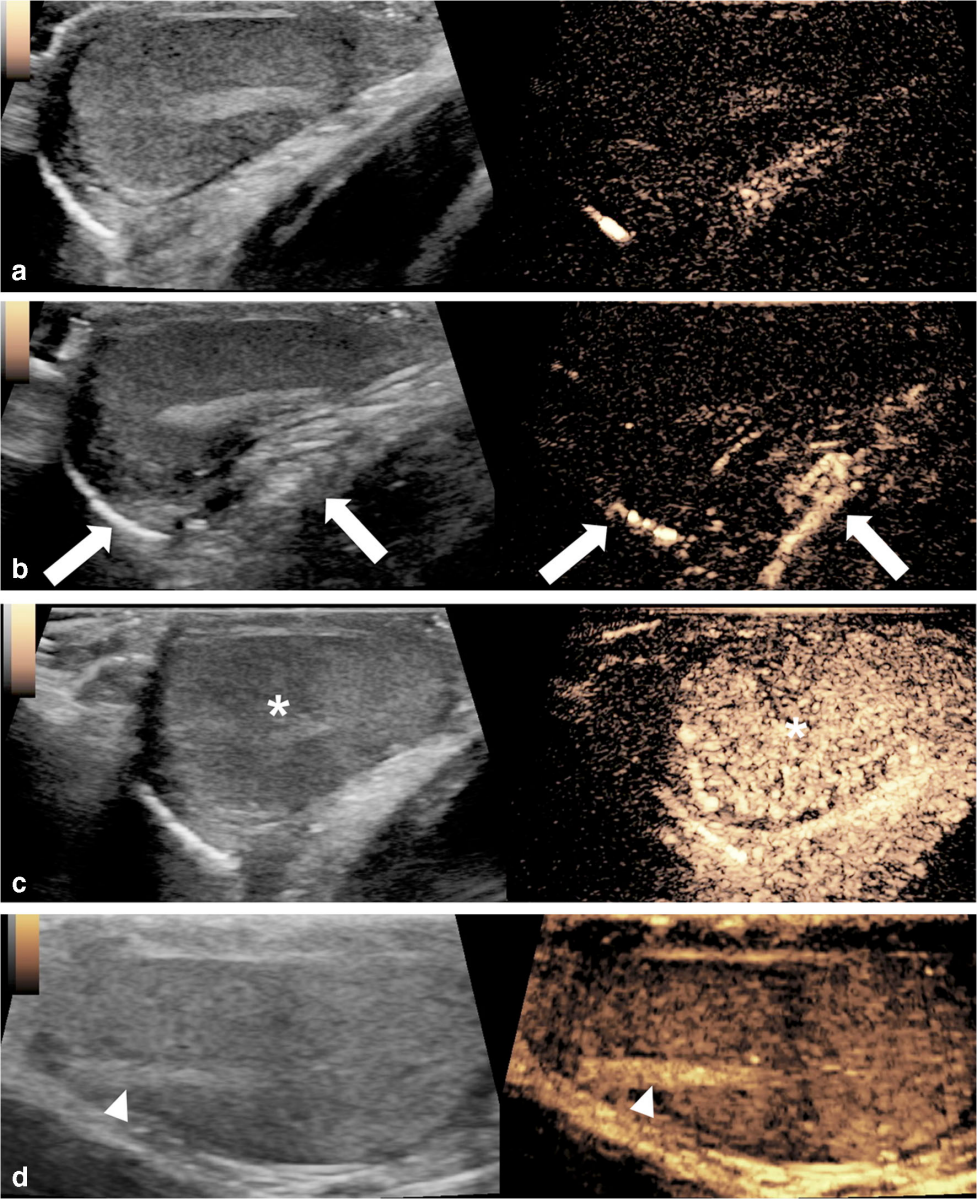
Advanced puberty in an 11-year-old boy undergoing high-resolution contrast-enhanced ultrasound (CEUS) of the right testis to assess for tumor. Normal testicular perfusion. **a–d** Transverse CEUS image with dual display of contrast (*right*) and gray-scale (*left*) modes. Two seconds post contrast injection (**a**), no intratesticular enhancement is visible. Eleven seconds post contrast injection (**b**), the first microbubbles appear in the scrotum and the testicular tunica (*arrows*). Twenty-two seconds post-contrast injection (**c**), there is homogeneous enhancement of the testicular tissue (*asterisk*). One hundred twenty seconds post-contrast injection (**d**), there is homogeneous enhancement of the testicular tissue including the mediastinum testis (*arrowhead*), without pathological changes

After the IV administration of the UCA, the testicular arteries enhance first. Within a few seconds there is uniform enhancement of the testicular parenchyma, which typically fades within 2–3 min [47]. In pre-pubertal boys, the testes are small and have relatively low flow, which can be difficult to detect by Doppler US. CEUS is more sensitive to slow velocity and low-volume blood flow and could provide a more conclusive demonstration of normal testicular parenchymal perfusion than color/power Doppler US (Fig. 4).

### Torsion

**Fig. 5:**
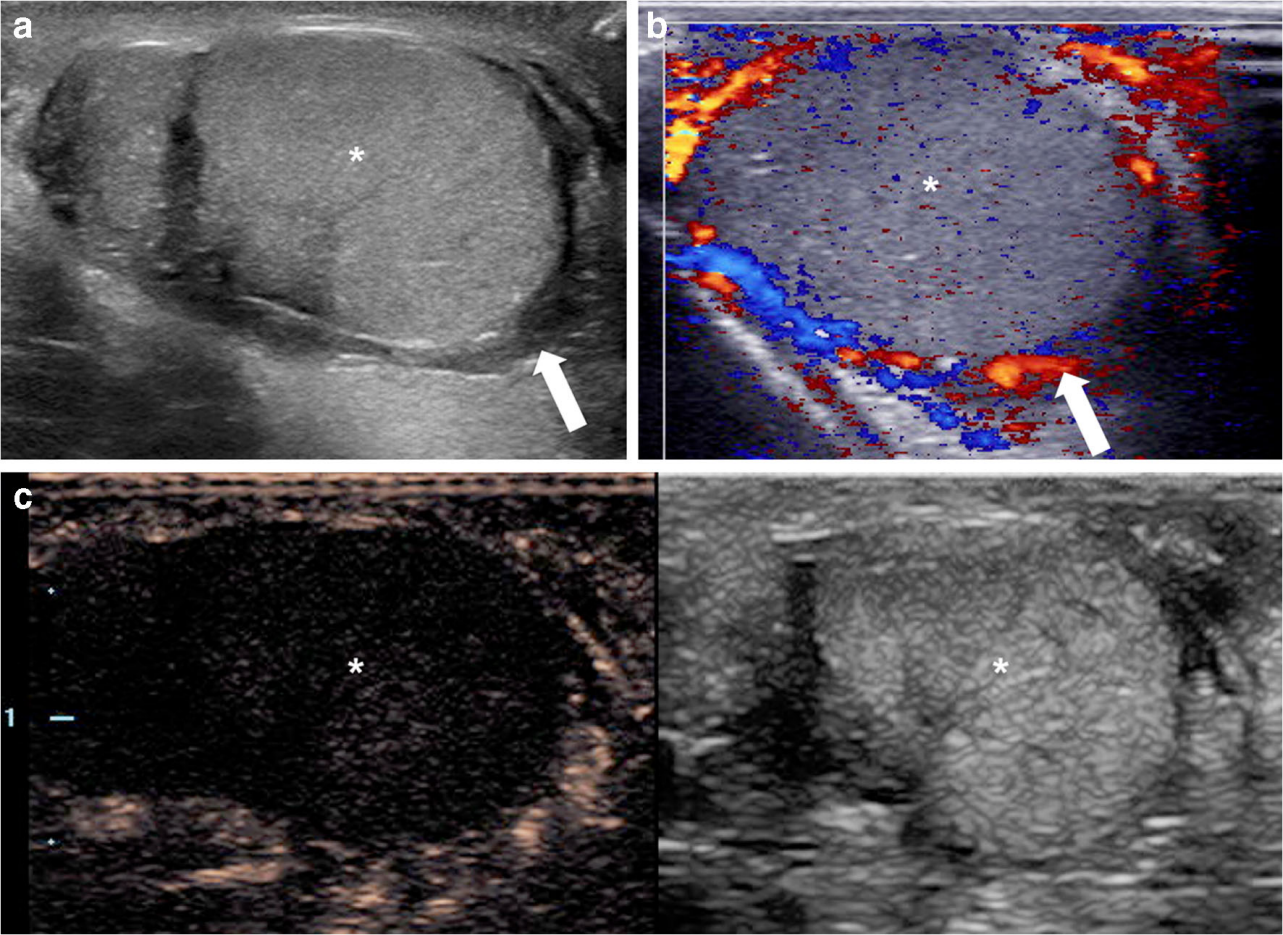
Acute scrotal pain in a 6-year-old boy with intellectual disability. **a** Longitudinal gray-scale US of the right testicle shows mild diffuse heterogeneity of the testicular parenchyma (*asterisk*). There is thickening and an edematous appearance of the scrotal wall (*arrow*). **b** Transverse color Doppler US image of the right testicle shows absence of color flow signal within the testicular parenchyma (*asterisk*). There is a rim of peripheral enhancement (*arrow*) corresponding to hyperemia of the scrotal wall. **c** Longitudinal dual display of contrast (*left*) and gray-scale (*right*) modes of the right testicle 49 s following contrast administration. There is complete absence of enhancement within the testicular parenchyma (*asterisk*), while contrast agent is seen in the surrounding tissues. See Online Supplementary Material 1 for cinematic images

**Fig. 6:**
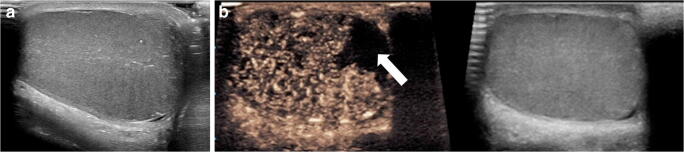
US in a 16-year-old-boy with severe pain of the left testicle. **a** High-resolution longitudinal gray-scale US of the left testicle shows homogeneous echogenicity with slightly coarse echotexture. **b** Longitudinal contrast-enhanced ultrasound (CEUS) with dual display of contrast (*right*) and gray-scale (*left*) modes, 21 s after contrast administration, shows a peripheral wedge-shape area of nonenhancement in the ventral segment of the lower pole of the left testicle, consistent with segmental infarction (*arrow*). Image reprinted with permission from [3]

When evaluating pediatric patients with acute-onset testicular pain, testicular torsion should always be considered, especially in adolescents [49]. A reported series for the assessment of acute testicular torsion did not demonstrate a clear advantage of CEUS over conventional color Doppler US [50]. However, CEUS might be helpful for problem-solving when it is clinically suspected that torsion has been missed in young children with small testes. In this case, CEUS might be used to confirm global testicular infarction by showing the absence of parenchymal enhancement of the entire affected testis (Fig. 5; Online Supplementary Material 1). In addition, CEUS might have a role in evaluating segmental infarction, which can be barely detectable on conventional gray-scale and color Doppler US in the early phase and has variable appearance as it evolves, making it a challenging diagnosis to differentiate from a tumor [51]. A segmental infarct appears as a focal, usually wedge-shape non-enhancing abnormality on CEUS (Fig. 6) [3, 52].

### Orchitis

In the setting of inflammation, CEUS can be valuable for evaluating more severe cases of epididymo-orchitis, especially when assessing for abscess or venous infarction. On CEUS, an abscess might exhibit a rim of increased peripheral enhancement with an absence of internal enhancement. Similar to other modalities, the lesion does not follow lobar distribution, which might help to differentiate this from a segmental infarction [46]. Extra-testicular epididymal abscesses demonstrate similar appearances [53].

### Focal testicular lesions

**Fig. 7:**
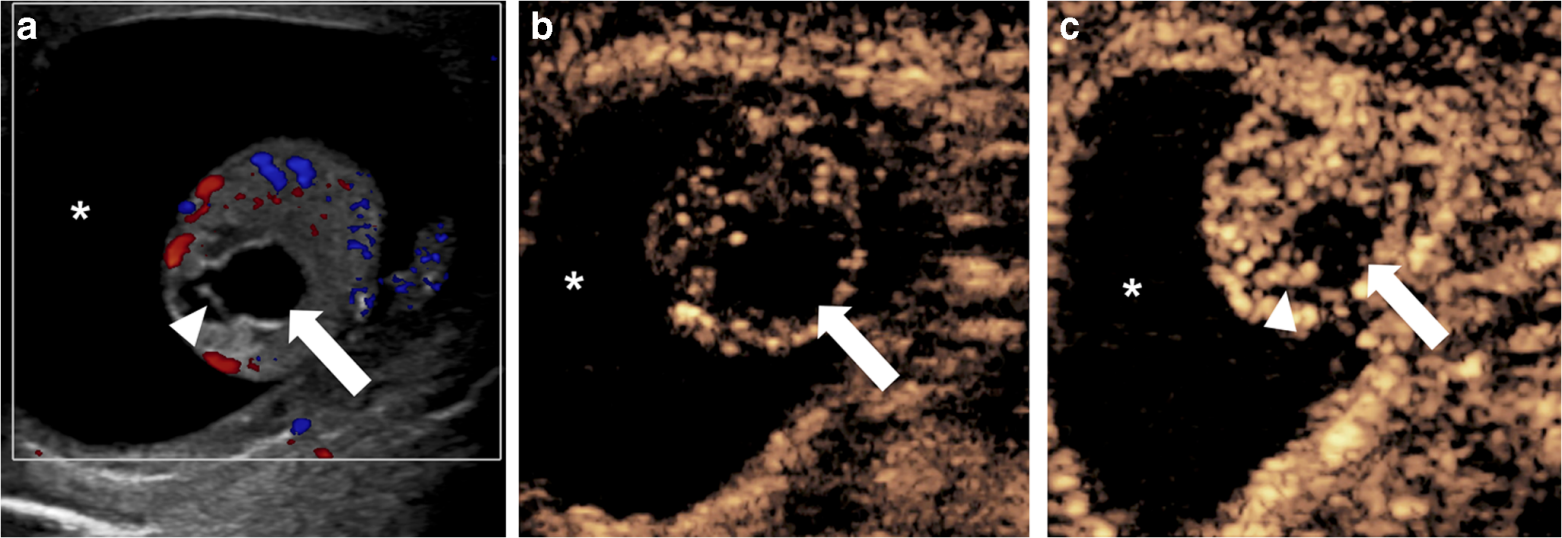
Left scrotal swelling in an 8-week-old boy. US was initially performed for evaluation of the swelling. An intratesticular cystic lesion was incidentally detected. **a** Transverse color Doppler US image of left testis. The testicular volume is normal for age. There is a large left-side anechoic hydrocele (*asterisk*). A cystic lesion (*arrow*) is noted centrally within the left testis, with a thin internal septum noted in the periphery of the lesion (*arrowhead*). **b, c** Transverse contrast-enhanced ultrasound (CEUS) of the left testis in contrast-only mode; 27 s post-contrast injection (**b**), there is absence of enhancement of the cystic lesion (*arrow*), and 44 s post-contrast injection (**c**), there is enhancement of the thin septum in the periphery of the lesion (*arrowhead*) that appears to be to the same extent as the surrounding normally enhancing testicular tissue. No evidence of enhancing nodularity is noted. There is no enhancement of the hydrocele (*asterisk*). Partial orchiectomy was performed, revealing changes of acute orchitis with hemosiderin deposition suggestive of sequala of previous inflammation

**Fig. 8:**
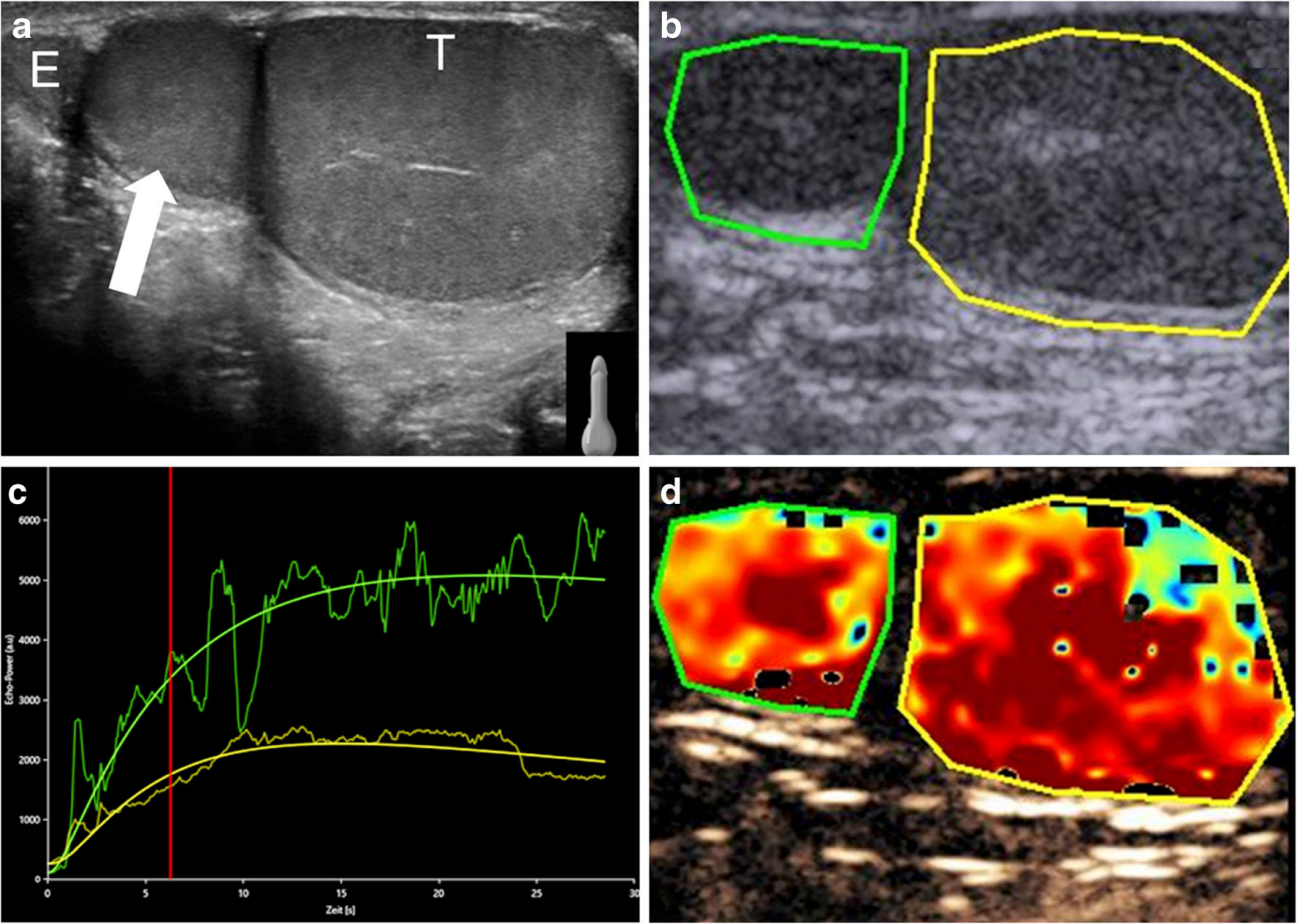
Palpable lesion adjacent to the right testicle in an 11-year-old boy. **a** Longitudinal gray-scale US of the right testis shows a round extra-testicular structure (*arrow*) between the epididymis (*E*) and the right testis (*T*), with similar reflectivity as the adjacent testicular tissue. **b, c** Quantitative contrast-enhanced ultrasound (CEUS) with time-intensity curve analysis, with a region of interest placed within the right testis (*yellow circle* in **b**) and the structure of concern (*green circle* in **b**). Time-intensity curve analysis of contrast dynamics (**c**) shows a similar wash-in and curve progression in the first 30 s for both structures. **d** Transverse CEUS color-coded map shows similar enhancing parameters for the structure of concern and the right testis. This combination of imaging findings favors the diagnosis of supernumerary testis compatible with polyorchidism

The task of differentiating between benign and malignant vascular abnormalities remains a challenge for all imaging techniques. CEUS offers the potential to improve diagnostic confidence when characterizing blood flow to intratesticular focal lesions, especially when the lesion is small [46, 54]. The lack of internal enhancement on CEUS is common for benign lesions such as a simple or an epidermoid cyst. This finding can increase examiner confidence that a lesion is benign (Fig. 7) [46]. If CEUS shows the presence of enhancement within a focal testicular lesion, this should be interpreted as concern for malignancy and requires further clinical and imaging interrogation [46, 48]. Several investigations attempted applying quantitative CEUS analysis of time-intensity curves to differentiate between malignant and benign intratesticular lesions [17–19]. In conjunction with gray-scale US findings, preliminary studies describing parameters such as wash-in rate, peak enhancement, time-to-peak and washout time are promising to distinguish benign from malignant lesions; however, others have reported similar time-intensity curves between these groups and therefore further studies are required in this field (Fig. 8).

Notwithstanding the current scarcity of published research on testicular applications, CEUS adds to the established sonographic evaluation of testes with its ability to provide a definitive assessment of vascularization. Thus, CEUS could increase examiner confidence and improve therapeutic strategies and timely management for the testicular pathology common in the children and adolescents.

## Female pelvis

Although CEUS is increasingly used to improve the specificity of US in many patient care settings and for many organs, there is a paucity of literature regarding CEUS of the female pelvis, with more limited studies specific to the pediatric population. Current guidelines do not include specific recommendations for the use of CEUS in female pelvis applications [1].

### Ovarian torsion

Ovarian torsion can occur at any age. This is when the ovary twists on its vascular and ligamentous supports, resulting in compromised blood supply. The presence of large cysts or other ovarian lesions, and large ovarian size predispose the ovary to twist; at least one of these factors is present in almost 75% of torsion cases [55]. In children, a possible predisposing factor is when the child has relatively long fallopian tubes and suspensory ligaments with resulting increased mobility of the ovary [55]. The most common US finding in ovarian torsion in pediatric patients is enlargement of the twisted ovary [56]. Other findings include peripheralization of ovarian follicles, a mass lesion within the twisted ovary, and twisting of the vascular pedicle or “whirlpool sign.” While using combinations of sonographic signs, gray-scale US has been shown to have specificity up to 87.5% for diagnosing ovarian torsion, whereas color Doppler US has been shown to have relatively limited sensitivity and specificity in confirming the diagnosis [57–59].

Using CEUS to evaluate ovarian torsion is promising. However, the normal enhancement characteristics of the ovary are not well described. In adults, CEUS has been shown to identify the “whirlpool sign” of the twisted vascular pedicle [60]. Absent or decreased perfusion is also seen at CEUS, with areas of necrosis and hemorrhagic infarction clearly demarcated [60]. A single pediatric case described the CEUS appearance of ovarian torsion and follow-up in an 6-year-old girl after laparoscopic untwisting and hyperbaric oxygen therapy [2]. This report included qualitatively decreased ovarian perfusion immediately following de-torsion, followed by hyperperfusion and a subsequent return to normal enhancement. Further experience might be needed before widespread clinical application.

### Ovarian masses

**Fig. 9:**
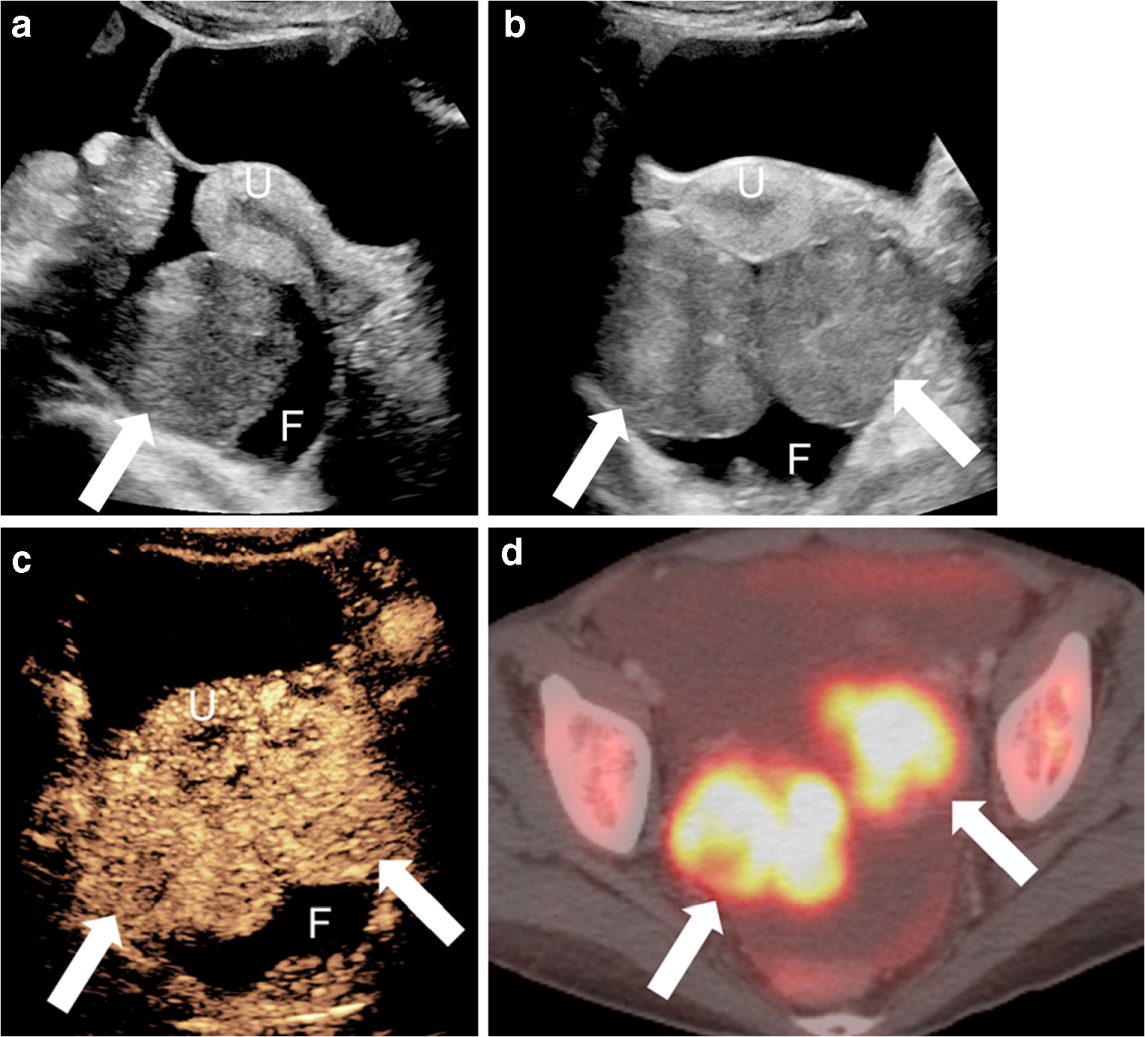
Imaging in an 18-year-old woman with cystic fibrosis status post lung transplant 4 years prior; she had a mediastinal mass concerning for post-transplant lymphoproliferative disease (PTLD). **a, b** Sagittal (**a**) and transverse (**b**) trans-abdominal gray-scale US demonstrates soft-tissue masses (*arrows*) posterior to the uterus (*U*) and small volume of free fluid (*F*) within the pelvis. **c** Transverse contrast-enhanced ultrasound (CEUS) of the pelvis in contrast-only mode at 2 min 30 s after contrast administration demonstrates heterogeneous enhancement of both ovarian masses (*arrows*), with no normal ovarian follicles visualized. **d** Axial positron emission tomography (PET)/CT demonstrates marked [F-18]2-fluoro-2-deoxyglucose (FDG) avidity of both ovarian masses (*arrows*), compatible with PTLD

**Fig. 10:**
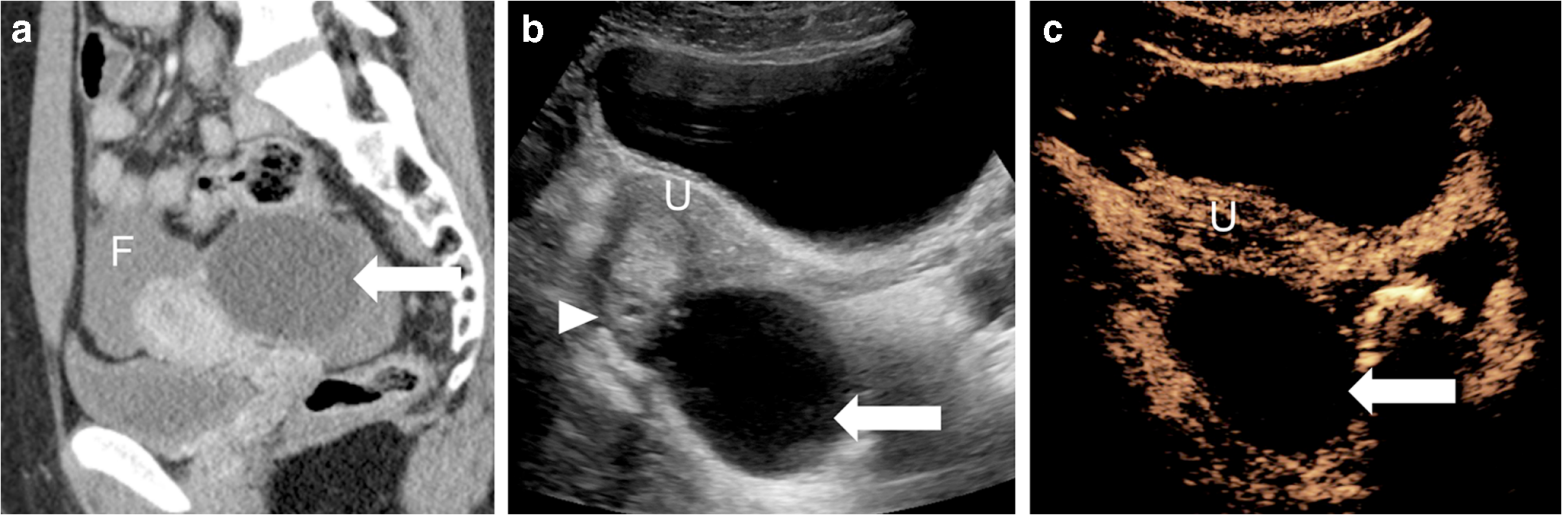
Ovarian cyst in a 16-year-old girl. CT was performed following blunt abdominal trauma. **a** Sagittal reconstruction from contrast-enhanced CT of the abdomen and pelvis demonstrates hemorrhagic fluid (*F*) in the pelvis from a splenic laceration (not shown here). A large cystic lesion (*arrow*) is noted posterior to the uterus. **b** Sagittal trans-abdominal gray-scale US demonstrates the anechoic lesion (*arrow*) posterior to the uterus (*U*) in direct contact with the right ovary (*arrowhead*). **c** Sagittal contrast-enhanced ultrasound (CEUS) of the pelvis in contrast mode shows no visible enhancement, nodularity or septations within the ovarian lesion (*arrow*), compatible with simple cyst

Contrast-enhanced US was shown to slightly improve the detection of ovarian cancer in adults, typically using a transvaginal approach. A meta-analysis published in 2016 reported sensitivity (0.97) and specificity (0.92) of CEUS for ovarian malignancy as higher than either gray-scale US (0.92 and 0.86) or Doppler US (0.93 and 0.85) assessment, respectively [61]. The heterogeneous enhancement of solid tumors is more visible at CEUS than Doppler US [62]. Quantitative analysis using time-intensity curve and the vascular enhancement describe malignant ovarian lesions as having greater peak enhancement intensity, delayed washout, and overall more enhancement than benign lesions (Fig. 9) [62]. Benign lesions such as hemorrhagic cysts and endometriomas do not enhance because these lesions have no internal perfusion and therefore can be distinguished with CEUS (Fig. 10) [63]. However, among these studies, some authors reported overlap of patterns between benign and malignant lesions such that a general threshold to differentiate one from the other cannot be established. Because of this, there are no current clinical recommendations for gynecological indications [1]. Extrapolating this evidence and applying it to the pediatric population is challenging because ovarian cancer comprises only 1–2% of all childhood malignancies, and ovarian tumors in adults differ from those in children, with the most common tumors in children being of germ cell origin [64]. No studies have evaluated CEUS in pediatric ovarian tumors.

### Endometrial hyperplasia and polyps

When using CEUS to image the uterus, typically the uterine artery enhances first, followed by the outer myometrium, inner myometrium and endometrium [62]. The endometrial peak enhancement is less than that of the myometrium on CEUS and washout time is longer than what occurs in the myometrium.

In the setting of endometrial hyperplasia, the endometrium enhances similar to the normal endometrium. On the other hand, endometrial polyps have more rapid arterial enhancement and delayed washout when compared to the endometrium and endometrial hyperplasia [62]. Adult studies have evaluated the use of CEUS in endometrial carcinoma; however, this disease does not occur in the pediatric population [65].

### Adenomyosis and leiomyoma (fibroid)

**Fig. 11:**
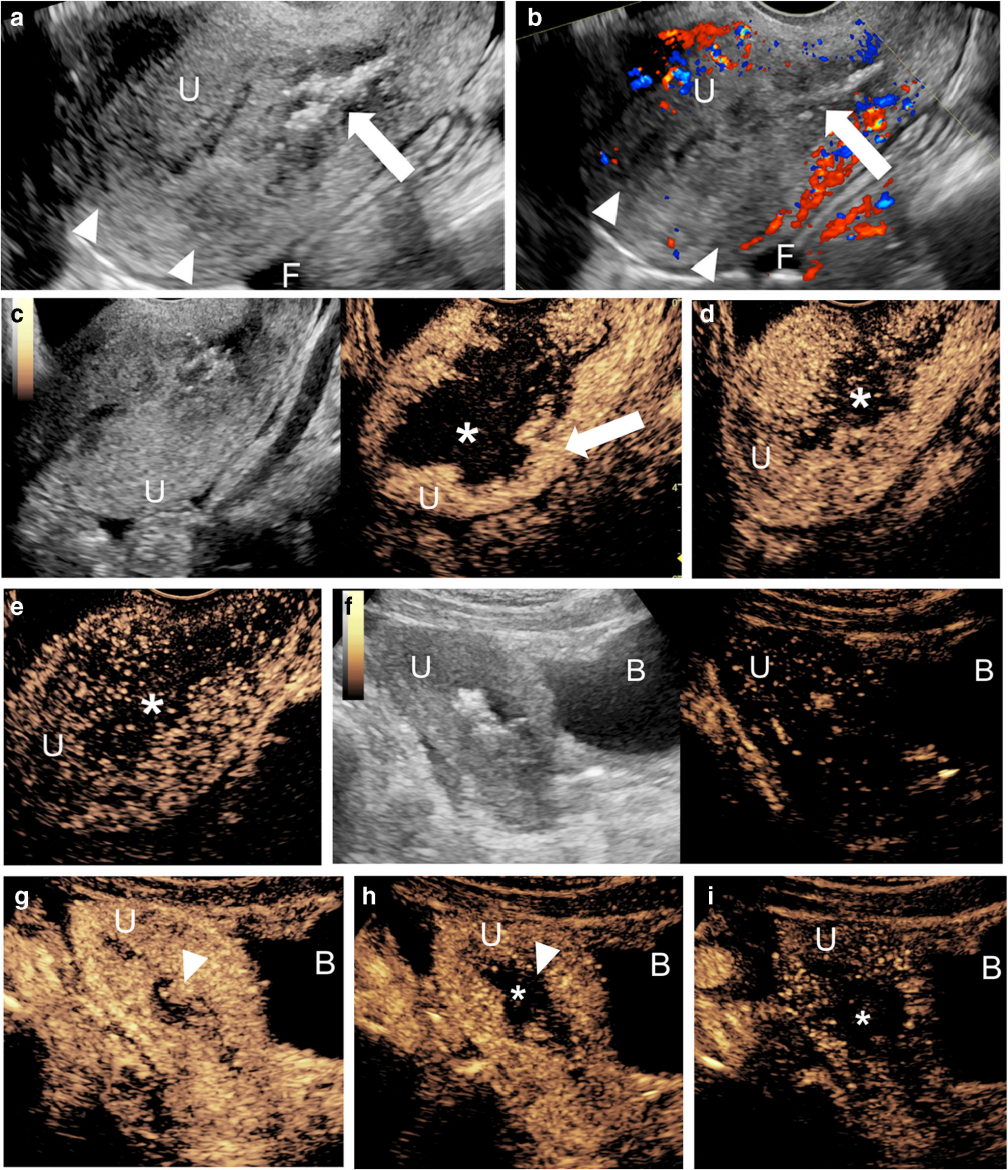
Imaging of a 16-year-old girl with vaginal bleeding. A fibroid lesion in the anterior wall of the uterus was biopsied hysteroscopically and had a pathological diagnosis of embryonal rhabdomyosarcoma. The girl was now presenting for hysteroscopic tumor debulking. **a** Intraoperative transvaginal gray-scale pelvic US, sagittal plane, shows that the uterus (*U*) is anteverted and demonstrates heterogeneous and poorly defined endometrium with multiple echogenic foci in the endometrial canal (*arrow*), likely reflecting gas loculi from the procedure. A heterogeneous, predominantly hypoechoic mass-like lesion is present within the central portion of the uterus, extending to the myometrium (*arrowheads*). A small volume of free fluid (*F*) is present within the pelvis. **b** Intraoperative transvaginal color Doppler US, sagittal plane, shows no significant color Doppler flow within the hypoechoic central regions (*arrowheads*). Gas loculi are noted within the endometrial canal (*arrow*). A small volume of free fluid (*F*) is present within the pelvis. **c–e** Intraoperative transvaginal contrast-enhanced ultrasound (CEUS) of the uterus in sagittal plane with simultaneous display of gray-scale (*left*) and contrast (*right*) images (**c**) and in contrast-only mode (**d** and **e**). CEUS was performed in the operating room during hysteroscopy, immediately following tumor debulking to evaluate for the extent of residual disease. A bolus dose of 1 mL Lumason was injected. Still images from a cinematic clip obtained during gentle sweeping through the uterus (*U*) at 20 s (**c**), 44 s (**d**) and 155 s (**e**). Peripheral lobulated enhancement (*arrow*) of the uterus corresponds to the residual tumor with persistent central nonenhancement (*asterisk*), likely to represent blood products from tumor debulking. **f–i** Intraoperative transabdominal CEUS of the uterus was subsequently performed in sagittal plane, following repeat bolus injection of 1 mL Lumason with simultaneous display of gray-scale (*left*) and contrast (*right*) images (**f**) and contrast-only mode (**g–i**). Still images of a stationary cinematic clip obtained at 20 s (**f**), 30 s (**g**), 109 s (**h**) and 156 s (**i**) following contrast administration show enhancement in the periphery of the uterus (*U*) with a lobular configuration and washout of the central tissue (*arrowhead*). Within the central portion of the uterus, there is persistent nonenhancement (*asterisk*), in keeping with blood products from tumor debulking. *B* bladder

Although uncommon in the pediatric setting, abnormal uterine bleeding can be caused by fibroids, adenomyosis or rare malignancies like rhabdomyosarcoma (Fig. 11). At US, fibroids are typically hypoechoic compared to the myometrium and have a pseudocapsule as well as possible areas of calcification. Unlike fibroids, adenomyosis usually has ill-defined margins [66]. However, distinguishing between the two at US can be challenging and CEUS might help to make a more accurate distinction. Fibroids tend to have peripheral capsular enhancement with gradual centripetal filling and diffuse enhancement in later phases. A feeding capsular vessel can often be seen. Necrotic fibroids show no central enhancement [67]. Conversely, adenomyosis has more rapid, diffuse, heterogeneous enhancement, with a “moth-eaten” appearance. No studies have specifically evaluated either of these two entities in the adolescent population.

## Conclusion

Similar to many advancements in medicine, the application of CEUS in adults is ahead of those for children. When it comes to the small organs, despite the differences in pathologies between children and adults, the experience gained from adult CEUS imaging is crucial for applying its merits in children. Therefore, by extrapolating data obtained from some CEUS examinations in adults, we can potentially reduce the delay in implementing CEUS in routine practice for children when imaging the small organs. CEUS is a promising application for these organs in children to assess perfusion and likelihood of malignancy.

## Supplementary Information

Online Supplementary Material 1 A 6-year-old boy with intellectual disability presents with acute scrotal pain. Same boy as in Fig. 5. Cinematic clip of the right testicle in longitudinal plane with dual display of contrast (left) and gray-scale (right) modes (MP4 44,886 kb)
